# Fungal Δ9-fatty acid desaturase: a unique enzyme at the core of lipid metabolism in *Aspergillus fumigatus* and a promising target for the search for antifungal strategies

**DOI:** 10.1128/mbio.00803-24

**Published:** 2025-06-26

**Authors:** Jonatas Erick Maimoni Campanella, Leonardo Talachia Rosa, Iran Malavazi

**Affiliations:** 1 Departamento de Genética e Evolução, Centro de Ciências Biológicas e da Saúde, Universidade Federal de São Carlos (UFSCar) 67828 , São Paulo, Brazil; 2 Department of Biochemistry and Tissue Biology, Biology Institute, Universidade Estadual de Campinas (UNICAMP) 28132, São Paulo, Brazil; 3 National Institute of Science and Technology in Human Pathogenic Fungi, São Paulo, Brazil; Instituto Carlos Chagas, Curitiba, Brazil

**Keywords:** *sdeA*, Δ9-fatty acid desaturase, *OLE1*, unsaturated fatty acid, *Aspergillus fumigatus*, antifungals, cytochrome b5

## Abstract

*Aspergillus fumigatus*, *Candida albicans*, and *Cryptococcus neoformans* are the leading fungal pathogens that cause life-threatening deep mycosis, posing significant challenges to immunocompromised patients and increasing healthcare costs worldwide. Lipid metabolism is crucial for the growth and development of all organisms. Increasing evidence highlights that complex structural lipids in the fungal cell membrane emerge as important factors involved in cell signaling, stress response, and immune recognition. Membrane fluidity is primarily regulated by the ratio of saturated and unsaturated fatty acids (UFAs), structural components of membrane phospholipids, and sphingolipids, which comprise UFAs with varying degrees of unsaturation. A notable group of UFA found in these molecules contains a *cis* double bond located at the C9 position of the carbon chain. The synthesis of such molecules is dependent on Δ9-fatty acid (FA) desaturase enzymes. In the absence of Δ9-FA desaturase, fungal cells become auxotrophic for palmitoleic and oleic acids (C16 and C18 UFA, respectively), suggesting that this essential enzyme family is fundamental for fungal physiology and virulence. However, the extent of phenotypes and especially the biochemical properties of fungal Δ9-FA desaturases remain poorly understood. In this manuscript, we summarize the current information and fundamental findings on Δ9-FA desaturase, gathered from functional studies on relevant fungal pathogens with a focus on *A. fumigatus* or deduced from model organisms, including yeasts and their mammalian counterparts. We also discuss its unique domain organization and its implications for the catalytic mechanism and the potential of fungal Δ9-FA desaturase as a chemotherapeutic target.

## FUNGAL INFECTIONS AND CURRENT TREATMENT CHALLENGES

Fungal infections pose a pervasive global health challenge, constituting a continuous and severe threat to human health. Recent estimates suggest that annually, over 6.5 million individuals grapple with invasive fungal infections, resulting in a staggering 3.8 million deaths worldwide (1–4). The landscape of fungal diseases has undergone significant transformations in recent decades, fueled by the emergence of critical pathogens like *Candida auris* and the re-emergence of well-recognized pathogens driven by the recurrent identification of antifungal-resistant strains worldwide (3, 5). Additionally, the COVID-19 pandemic contributed to reshaping the epidemiology of these diseases (6, 7).

*Aspergillus* species, especially *Aspergillus fumigatus,* take center stage as the primary filamentous fungi responsible for most cases collectively known as aspergilloses. The spectrum of *A. fumigatus*-related diseases encompasses life-threatening invasive pulmonary aspergillosis (IPA) in immunosuppressed individuals, along with a range of other clinical manifestations diagnosed in immunocompromised, atopic, and immunocompetent patients (reviewed in reference 8). As a result, these elevated mortality and morbidity rates pose not only challenges to the clinical management of the patients but also related to the costs associated with the treatment (9, 10).

As the cell wall and cell membrane constitute the first barriers between the pathogen and the environment of the host organism, they stand out as important fungal therapeutic targets. There are three main clinical antifungal drugs: (i) polyenes, the oldest antifungal drug class, which includes amphotericin B (AMB); (ii) azoles (e.g., voriconazole); and (iii) echinocandins, the newest class of antifungals comprising, for instance, caspofungin whose antifungal activity relies on the noncompetitive inhibition of β-1,3-glucan synthase. Although echinocandins boast reasonable safety profiles and lower toxicity, their high cost and short half-life limit their usage (11, 12). In addition, echinocandins exhibit fungistatic activity against *A. fumigatus* and are generally not recommended as monotherapy for IPA, positioning them as a second-line therapeutic option (13, 14).

Both azoles and polyene molecules target the cell membrane. AMB binds the fungal-specific sterol (ergosterol) accumulated in the plasma membrane, thus causing ergosterol sequestration and the formation of concentration-dependent channels that allow ions and the intracellular content to leak, causing cell death (15). AMB has long been the standard therapy for serious fungal infections despite concerns about the manifestation of acute renal failure (13, 16, 17). Although lipid formulations of AMB (e.g., liposomal AMB) retain lower renal toxicity and dysfunction than the original formulation with deoxycholate, they have substantially higher costs that limit wide use (18, 19). Azoles inhibit ergosterol production by acting on the cytochrome P450 enzyme sterol 14α-demethylase, an endoplasmic reticulum (ER) enzyme that converts lanosterol to ergosterol, named Cyp51 in molds and Erg11 in yeasts (20, 21). Inhibition of 14α-demethylase is fungistatic in yeasts and fungicidal in molds. Therefore, azoles represent the clinically most relevant subgroup of drugs and the choice therapy for treating IPA due to their high effectiveness, low toxicity, immunomodulatory capacity, and oral application feasibility (12, 22).

In the last decades, the number of fungal clinical isolates resistant to some of these antifungals has dramatically increased, representing one of the challenging obstacles in combating fungal diseases for the upcoming years (20). Epidemiological surveillance studies report significant azole resistance in *A. fumigatus* (23). Chief among the causes that favor the worldwide emergence of azole-resistant *A. fumigatus* strains is the extensive use of agricultural fungicides for crop protection, which are chemically related to antimycotics belonging to the triazoles group (18, 24–26) and the prophylactic and/or long-term azole treatment regimens in patients (23). Similarly, resistance to polyenes within *Aspergillus* species has increased in the past decade (27). A meta-analysis report suggested that 2.01% of 17,494 *A*. *fumigatus* isolates were AMB resistant (28).

Lastly, fungal biofilms can confer resistance to most clinically available drug classes. In the last decade, active research has been conducted to understand how biofilm formation impacts drug resistance and availability. To this end, some genetic, morphological, and microenvironmental factors have been identified in filamentous fungal biofilm biology that significantly impact the ability of commonly used drugs to act on a mature mold biofilm (29–31).

While the existing clinical antifungal drugs belonging to the abovementioned drug classes continue to provide significant contributions to antifungal chemotherapy, the limitations imposed by resistance development, clinical complexities associated with every form of fungal infection, and the unique lifestyle of filamentous fungi versus yeasts highlight the critical need for innovative solutions. Considering these challenges and the ever-evolving landscape of fungal diseases, searching for new targets that could be translated into new antifungal molecules is an important strategy to advance medical mycology. Nonetheless, approximately 80% of antifungal targets in the literature are false positives with little potential to develop target-based inhibitors with desirable features (32). This is primarily because the human host and the invading fungal pathogens share significant similarities in their eukaryotic cell machinery, which governs nearly all the cellular processes. As a result, toxicity remains a persistent concern. However, targeting fungal essential genes shared with the host, particularly those encoding enzymes required for establishing or sustaining infection, offers a promising strategy for identifying novel chemotherapeutic targets, provided these enzymes exhibit some degree of functional differences in their catalytic mechanisms.

Recent studies have linked enzymes involved in lipid metabolism to fungal virulence and development (33–39). However, their potential as drug targets remains uncertain, as does the understanding of how the intricate series of biochemical processes involving fungal enzymes and transporters—responsible for the *de novo* synthesis, degradation, or uptake of lipids—impact cell membrane composition, signaling pathways, or lipid-dependent host immune mechanisms (reviewed in reference 40). An elegant demonstration of this scenario comes from *C. neoformans* literature, where the unsaturated fatty acid (UFA) oleic acid resident in lipid droplets enhances the fungal replication rate of both intracellular and extracellular *C. neoformans*, indicating the ability of the fungus to access and utilize fatty acids (FA) from lipid droplets within macrophages. Furthermore, exogenous oleic acid supplementation to cultured macrophages significantly increased rates of nonlytic exocytosis of the fungal cells from macrophages, key players in the immune system (41).

This pivotal requirement for oleic acid, which serves as a precursor for building complex fungal membrane lipids, sets the stage for dynamic control of the *de novo* UFA synthesis, where the maintenance of fungal membrane homeostasis, composition, and fluidity becomes a central theme. This review article explores the multifaceted aspects of the fungal ∆9-FA desaturases as potential drug targets for fighting fungal infections. ∆9-FA desaturases are essential for the viability of many fungal pathogens (35, 38, 42), responsible for the conversion of the saturated fatty acids (SFA), palmitic and stearic acids, into their monounsaturated counterparts, palmitoleic and oleic acids, respectively. Although the desaturation of these SFA occurs in a single reaction, it comprises multiple steps of electron transfer that highlight the complexity of this enzymatic mechanism that culminates with the formation of a *cis* double bond and water (43–46).

**Fig 1:**
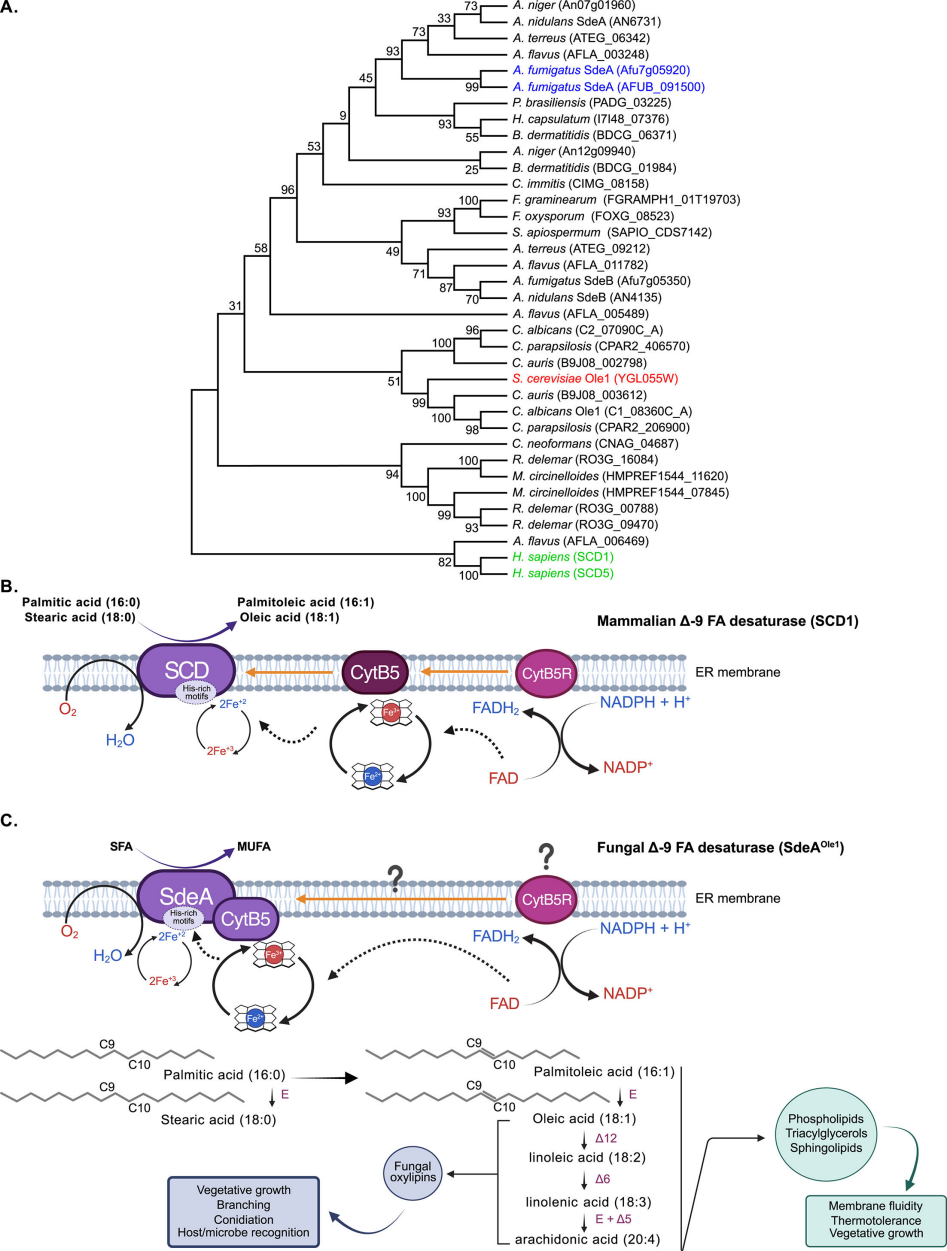
Comparative phylogenetic and biochemical model of mammalian stearoyl-CoA desaturases and fungal SdeA^Ole1^ desaturases. (**A**) Phylogenetic relationship of Δ9-FA desaturase in homologous mammals and selected pathogenic fungi. Maximum likelihood tree based on protein sequences from fungi and humans reveals distinct clades for SdeA and SdeB homologs, yeast Ole1 enzymes, and human SCD1 and SCD5 outgroup. Bootstrap values (based on 1,000 replicates; Mega11 software) are indicated at the nodes. (**B**) Scheme of the unsaturated fatty acid biosynthetic pathway in mammals (SCD1) (**C**) and the proposed model for fungal (SdeA^Ole1^) desaturase. The orange arrows indicate the necessity of direct interactions between the desaturase (SCD1), CytB5, and cytochrome B5 reductase (CytB5R). The dotted arrows represent the electron flux during the desaturation process. Reduced and oxidized molecules and iron atoms (Fe^2+^ and Fe^3+^) are shown in blue and red, respectively. The histidine-rich motifs comprise HXXXXH, HXXHH, and QXXHH, which contain the His residues that coordinate a nonheme di-iron center within the catalytic site of the desaturase domain and are essential for catalysis. The CytB5 domain contains the heme iron center represented by the simplified heme structure where the iron atoms are coordinated and cycle between Fe^2+^ and Fe^3+^ during the electron transfer. Question marks indicate that the CytB5R is not identified in fungi. MUFA, monounsaturated fatty acid; E, elongases; Δ12, Δ6, Δ5, desaturases that introduce double bonds at carbons C12, C6, or C5 of the fatty acid acyl chain. Created in BioRender (https://BioRender.com/w1ilztm).

Building on our previous contributions, we provide evidence about the unique biochemical and structural features of the ∆9-FA desaturase SdeA (stearic acid desaturase) of *A. fumigatus* as a prototype of all fungal enzymes belonging to this group of desaturases. We emphasize the observation that the biochemical reaction catalyzed by this enzyme depends on the C-terminal cytochrome B5 (CytB5) domain, which serves as the electron donor source for the enzyme’s desaturase domain—that harbors the catalytic site—to introduce a double bond in natural substrates of this enzyme. A review of the literature and in-depth sequence reanalysis revealed that the desaturase domain of these enzymes is expressed as a fusion protein together with the CytB5 domain, naturally present in fungal ∆9-FA desaturases. This organization sharply contrasts with human orthologs, where the electron donor source is provided by a separate CytB5 polypeptide, allowing the desaturation of palmitic and stearic acids (Fig. 1). Given this exclusive structural organization, we propose that in addition to inhibiting the enzyme’s catalytic site to disrupt its activity, targeting the unique fusion CytB5 domain in fungal ∆9-FA desaturases provides a novel approach for modulating this essential enzyme in fungal pathogens. This strategy holds significant promise for controlling fungal growth by exploiting the structural and functional peculiarities of this enzyme.

## TARGETING THE FUNGAL CELL MEMBRANE BEYOND ERGOSTEROL: UNSATURATED FATTY ACID SYNTHESIS ON FOCUS

The composition of fungal cell membranes shows some variations among different species, but as in all eukaryotic cells, it typically includes different groups of phospholipids (PL) and sphingolipids (SL) as primary building blocks. These lipid molecules, along with ergosterol, are spatially organized into a lipid bilayer that forms the fundamental structure of the plasma membrane. This bilayer comprises a hydrophilic phosphate head and one or more hydrophobic acyl tails. The proportions and spatial locations between these components play a critical role in maintaining membrane function and stability (47). Phosphatidylcholine and phosphatidylethanolamine are the main PL classes in fungal membranes, comprising the majority of the fungal phospholipid pool, including the filamentous fungi *A. fumigatus* and *Fusarium graminearum* (35, 48). The remaining belongs to anionic phospholipids: phosphatidylserines and phosphatidylinositols (49).

Collectively, the composition of PL and SL determines the fluid state and molecular order of the biological membranes. These molecules incorporated distinct FAs that attach to the glycerol carbons or the sphingoid base, respectively (35, 50). Membrane fluidity varies with temperature, as UFAs become disorganized and loosely packed, enhancing fluidity, while SFAs remain tightly packed due to their higher melting points, maintaining rigidity. As temperatures rise, both the degree of FA saturation and the length of FA chains in phospholipids increase (reviewed in reference 51). Consequently, the balance of SFA and UFA accounts for the remarkable chemical diversity of PL and SL, directly influencing the fate of the monounsaturated FA (MUFA) synthesized within the cell from SFA precursors via a single desaturation reaction catalyzed by Δ9-FA desaturase enzyme. The newly synthesized MUFA pool also serves as substrates for additional desaturation reactions, producing polyunsaturated FA (PUFAs), such as linoleic acid (18:2) through Δ12-FA desaturase activity and linolenic acid (18:3) via Δ6-FA desaturase. These PUFAs are further elongated to arachidonic acid (20:4) through elongases and additional desaturation catalyzed by Δ5-FA desaturases (Fig. 1B). Beyond their essential roles as components of PL and SL, PUFAs are critical for the biosynthesis of lipid-derived signaling molecules, such as oxylipins in fungi (52). Notably, fungal oxylipins are examples of molecules involved in intra- and intercellular communication, highlighting the importance of Δ9-FA desaturase as a key enzyme that provides MUFA precursors for oxylipin biosynthesis (Fig. 1C). In *A. fumigatus*, oxylipin 5,8-diHODE is important for cellular differentiation, lateral branching, and echinocandin tolerance (53–55).

Fungal Δ9-FA desaturases are ER-resident proteins commonly called *OLE1* (OLEic acid requiring) in yeast, such as *S. cerevisiae*, *C. albicans*, and *C. neoformans*. In filamentous fungi, such as *A. nidulans* and *A. fumigatus*, the corresponding enzyme is encoded by the *sdeA* gene and referred to as SdeA (35, 36, 56). These enzymes are highly conserved across fungal species but share only 40% sequence identity with the human stearoyl-CoA desaturase 1 (SCD1) and SCD5 isozymes (Table 1), as previously reported (35). This limited sequence identity can be attributed to significant differences in structural organization. While both fungal and mammalian Δ9-FA desaturases possess an N-terminal desaturase domain with a di-iron catalytic center, fungal Δ9-FA desaturases uniquely include a C-terminal CytB5 domain naturally fused to the desaturase domain. These unique features of fungal Δ9-FA desaturases will be further explored in the upcoming sections.

**TABLE 1:** Δ9-FA desaturases in pathogenic fungi identified through blast search analyses and comparison with human SCD1, *S. cerevisiae* Ole1, and *A. nidulans* SdeA and SdeB

Organism (strain)	Gene	%ID *Hs* SCD1	%ID *Sc* Ole1	%ID *Af* SdeA	%ID *An* SdeA	%ID *An* SdeB	CytB5 domain
*Homo sapiens*	SCD1	100	36.63	40.82	40.82	38.35	No
*Homo sapiens*	SCD5	60.63	33.57	40.43	38.30	39.21	No
*Saccharomyces cerevisiae* (S288c)	YGL055W (*OLE1*)	36.63	100	52.8	51.49	52.12	Yes
*Aspergillus fumigatus* (Afu293)	Afu7g05920 (*sdeA*)	40.82	52.80	100	91.89	54.46	Yes
*Aspergillus fumigatus* (Afu293)	Afu7g05350 (*sdeB*)^*b*^	28.44	40.41	54.46	53.58	59.88	No
*Aspergillus fumigatus* (A1163)	AFUB_091500 (*sdeA*)	40.82	52.80	100	91.89	54.46	Yes
*Aspergillus fumigatus* (A1163)	AFUB_090930^*a*^	nr^*c*^	nr	nr	nr	nr	nr
*Aspergillus nidulans* (A4)	AN6731 (*sdeA*)	40.82	51.49	91.89	100	69.43	Yes
*Aspergillus nidulans* (A4)	AN4135 (*sdeB*)	38.35	50.71	69.75	69.75	100	Yes
*Aspergillus flavus* (NRRL3357)	AFLA_003248	40.45	52.33	93.64	92.98	69.52	Yes
*Aspergillus flavus* (NRRL3357)	AFLA_011782	38.97	53.62	75.51	73.47	75.06	Yes
*Aspergillus flavus* (NRRL3357)	AFLA_005489	35.19	47.66	58.21	58.71	55.88	Yes
*Aspergillus flavus* (NRRL3357)	AFLA_006469	33.89	38.12	43.48	45.62	41.46	Yes
*Aspergillus niger* (CBS 513.88)	An07g01960	41.20	51.46	92.76	93.86	66.96	Yes
*Aspergillus niger* (CBS 513.88)	An12g09940	39.05	50.49	77.64	77.91	68.40	Yes
*Aspergillus terreus* (NIH2624)	ATEG_06342	40.07	53.05	92.76	92.31	69.02	Yes
*Aspergillus terreus* (NIH2624)	ATEG_09212	40.83	55.98	78.49	78.43	79.49	Yes
*Blastomyces dermatitidis* (ER-3)	BDCG_01984	40.82	51.84	89.40	85.96	68.79	Yes
*Blastomyces dermatitidis* (ER-3)	BDCG_06371	31.18	45.52	65.92	65.54	61.80	Yes
*Candida albicans* (SC5314)	C1_08,360C_A (*OLE1*)	37.50	58.30	56.20	56.97	55.16	Yes
*Candida albicans* (SC5314)	C2_07,090C_A	26.28	31.58	33.47	33.47	32.56	Yes
*Candida auris* (B8441)	B9J08_003612	37.97	59.66	57.29	57.21	55.06	Yes
*Candida auris* (B8441)	B9J08_002798	26.48	32.05	34.13	33.76	33.19	Yes
*Candida parapsilosis* (CDC317)	CPAR2_206900	36.73	58.42	55.99	57.00	56.17	Yes
*Candida parapsilosis* (CDC317)	CPAR2_406570	25.86	31.70	33.46	33.08	34.13	Yes
*Coccidioides immitis* (RS)	CIMG_08158	38.35	52.79	85.31	82.71	69.51	Yes
*Cryptococcus neoformans* (H99)	CNAG_04687	39.47	45.85	56.50	56.96	56.50	Yes
*Fusarium graminearum* (PH-1)	FGRAMPH1_01T19703	37.22	55.08	77.48	77.23	69.35	Yes
*Fusarium oxysporum* (f. sp. lycopersici 4287)	FOXG_08523	38.35	54.82	78.83	78.83	69.82	Yes
*Histoplasma capsulatum* (G217B)	I7I48_07376	39.33	53.05	87.72	85.53	68.05	Yes
*Mucor circinelloides* (1006PhL)	HMPREF1544_07845	40.64	46.37	52.20	52.20	55.29	Yes
*Mucor circinelloides* (1006PhL)	HMPREF1544_11620	38.60	46.41	51.07	51.82	53.70	Yes
*Paracoccidioides brasiliensis* (Pb18)	PADG_03225	39.70	51.56	88.18	84.43	70.11	Yes
*Rhizopus delemar* (RA 99-880)	RO3G_00788	38.16	44.99	53.66	51.95	56.08	Yes
*Rhizopus delemar* (RA 99-880)	RO3G_09470	35.71	45.38	53.98	52.44	56.08	Yes
*Rhizopus delemar* (RA 99-880)	RO3G_16084	39.62	43.75	52.92	53.54	51.24	No
*Scedosporium apiospermum* (IHEM 14462)	SAPIO_CDS7142	36.84	54.73	74.11	74.27	68.81	Yes

^*a*^ Annotated as a pseudogene in A1163 strain and nominated as sdeB (noncharacterized).

^*b*^ Annotated as a functional gene in Af293 strain (noncharacterized).

^*c*^ nr, not relevant.

### Regulation and function of Δ9-fatty acid desaturase in fungal biology

Although the regulation of Δ9-FA desaturase gene expression is of growing interest due to its relevance to various biological processes, the underlying mechanisms across different organisms remain incomplete. Studies have revealed convergent regulatory strategies spanning bacteria to humans. These conceptual convergent mechanisms involve the activation of signal transduction pathways triggered by alterations in the physical properties of the cell membrane, which subsequently regulate Δ9-FA desaturase gene expression. Second, the accumulation of oleic acid can act as negative signaling molecules, effectively shutting down the pathway (57–60).

In mammals, SCD1 expression is controlled by a signaling pathway that regulates trafficking between the ER and Golgi apparatus. This involves intramembrane proteolytic processing of the sterol regulatory element-binding protein (SREBP-1), which is sensitive to PUFA and cholesterol levels (57, 61). In the presence of PUFAs, the production of active, soluble, and nuclear SREBP-1 protein is repressed, reducing its transcriptional activity and consequently downregulating SCD1 expression (57). In *A. fumigatus*, the transcription factor SrbA, an SREBP ortholog, plays a critical role in adaptation to hypoxic conditions and iron uptake, both essential for fungal survival within the host environment (62, 63). Likewise, cleavage of full-length SrbA to generate the N-terminus of SrbA containing the bHLH domain, which localizes to the nucleus to regulate SrbA-targeted gene transcription, also occurs in *A. fumigatus* (64). However, the impact of SFA, UFA, or PUFA levels in the biochemical processing of SrbA or other fungal SREBP-1 ortholog has not been investigated so far.

Given that Δ9-FA desaturases, such as SdeA^Ole1^, require molecular oxygen, and reactions occur under aerobic conditions (43), it is reasonable that *sdeA* is regulated in response to oxygen and low iron availability. Since most oxygen-dependent enzymes are also iron containing, responses to iron starvation and hypoxia are often coordinately regulated (65, 66). This connection suggests that SdeA activity plays a significant role in maintaining the balance between SFA and UFA under hypoxic conditions. Therefore, SdeA activity likely safeguards the SFA/UFA balance under hypoxia, enabling fungal growth and proliferation during infection within the host environment. This is consistent with the regulation of mammalian glioblastoma cells in that SCD1 expression was elevated in hypoxic conditions in an SREBP-dependent manner (67). This regulatory mechanism appears to be conserved in *A. fumigatus*, as the *sde*A gene is downregulated under hypoxia in an *srb*A mutant (62).

Regulatory mechanisms for Δ9-FA desaturases and iron metabolism have been proposed. For example, in the insect pathogen *Beauveria bassiana*, the regulation of Ole1 is directly mediated by the basic leucine zipper (bZIP) transcription factor HapX. This regulation affects iron acquisition and conidial lipid reserves, the latter impacting the membrane functionality, potentially relevant in the early stages of *B. bassiana* infection (68). HapX was also extensively characterized as an important transcription factor involved in iron uptake and virulence in *A. fumigatus* (69, 70). Notably, HapX is part of the regulatory network that controls SrbA expression. Recently, *sdeA* was highlighted as co-regulated by both SrbA and HapX through a comparative analysis of ChIP-Seq data sets for these transcription factors (71).

Temperature is a key environmental factor influencing fungal pathogenesis, directly affecting cellular physiology, as it constantly fluctuates. Under these conditions, the cell membrane is likely the primary structure to detect temperature changes, undergoing significant alterations in its chemical composition, fluidity, and other physical properties, primarily through SFA/UFA ratio adjustments (72–74). Consistently, transcriptional upregulation of *OLE1* has been observed in *S. cerevisiae* in response to low temperatures to maintain the fluidity state of the membrane due to the increase in the number of MUFA and PUFA in the cell membrane (75). Similarly, the same response occurs in *C. albicans* and *A. fumigatus*. As expected, genetic depletion of *A. fumigatus sdeA*^OLE1^ largely reduced the composition of MUFAs (35, 76). Taken together, these observations reinforce the notion that temperature affects the fluidity of the membrane via the direct action of *OLE1* or *sdeA*.

When cells experience sublethal heat shock (HS), their biosynthesis shifts transiently, while the expression of heat shock protein (HSP) genes is activated so the cells can survive subsequent exposure to otherwise lethal temperatures or other environmental stresses (77). This response is mediated by the highly conserved heat shock transcription factor Hsf1, where orthologs have been characterized in several pathogenic fungi and are essential for viability (78, 79). The regulatory network that emerges from the Hsf1 response includes genes involved in cell proteostasis as well as energy and carbohydrate metabolisms, cell wall integrity, host recognition, and virulence (77, 79, 80). Notably, genetic depletion of *C. albicans OLE1* disrupts the heat stress response by impairing Hsf1 full activation during temperature upshift. This impairment occurs at both the transcriptional and protein levels, leading to reduced expression of HSP, such as *HSP104* and *HSP21,* along with lower phosphorylation of Hsf1 (76). Additionally, the E3 ubiquitin ligase Rps5, a key component of the ubiquitin/proteasome-dependent ER-associated degradation (ERAD) system, positively regulates *OLE1*, likely through its role in the release from the ER membrane of the soluble polypeptide of the transcription activator Spt23 that subsequently migrates to the nucleus and specifically controls the expression of *OLE1* in response to the UFA content and environmental stresses (58, 76, 81). Spt23 is relative to mammalian NF-κB (59). In *A. fumigatus, hsfA* depletion using a conditional *xylP::hsfA* mutant compromises SdeA expression, suggesting a positive role of HsfA over SdeA expression at basal (30°C) condition (35, 79). Furthermore, the HsfA-Hsp90 circuit was recently associated with the SdeA activity in *A. fumigatus* since Hsp90 and SdeA physically interact (35). Accordingly, *S. cerevisiae* Ole1 physically interacts with Hsp82 (82), which is part of the Hsp90 family.

Another example highlighting the role of UFAs in thermotolerance regulation is observed in the fungal pathogen *Histoplasma capsulatum*. The expression of *HSP82*, a chaperone belonging to the Hsp90 family, and *HSP70* genes is modulated by *OLE1* expression levels and by the supplementation of SFA or UFA in the culture medium (83, 84). During heat shock, supplementation with oleic acid reduces HS-responsive gene transcription. In contrast, the addition of palmitic acid induces the opposite effect, reinforcing the interplay between FA composition and HS gene regulation. In addition, treatment of *H. capsulatum* cells with a monoclonal antibody (mAb) against Hsp60, a surface protein involved in infection, revealed that UFA biosynthesis was one of the most over-represented pathways. Proteomic analysis revealed that mAb treatment reduced the levels of Δ9-FA desaturase by approximately 40% but increased the levels of the oleate Δ−12 desaturase, which was accompanied by increases in the level of PUFA (85). Altogether, these findings suggest that the cell membrane senses temperature via a mechanism involving oleic acid levels, Ole1^SdeA^ activity, and Hsf1^HsfA^ activation, although the underlying mechanisms are not fully elucidated.

In *C. albicans,* filamentation depends on the level of *OLE1* expression, impacting the formation of hyphal filaments and chlamydospores in aerobic conditions (38). A similar regulatory mechanism has been observed in the dimorphic fungal pathogen *Paracoccidioides brasiliensis*, where *OLE1* mRNA levels are correlated with the yeast-to-mycelia transition, although the exact mechanism remains unknown (86). Reduced *OLE1* expression in conditional mutants impairs the virulence of both *C. albicans* and *Candida parapsilosis* in murine infection models and decreases susceptibility to macrophage-mediated killing (42, 87). Furthermore, in *A. nidulans*, biochemical evidence suggests that UFA and their derivatives are critically involved in conidiation and the formation of multicellular developmental structures, including conidiophores, cleistothecia, and sclerotia (56, 88).

*A. fumigatus* SdeA overexpression exhibited impaired vegetative growth, itraconazole sensitivity, and altered lipid metabolism. Lipid analysis revealed decreased accumulation of palmitic and stearic acids and increased production of palmitoleic (~5.9-fold) and oleic (1.8-fold) acids, as well as some PUFAs, especially eicosadienoic acid (20:2; ~2.39-fold) (36). As expected, genetic depletion of *sdeA* resulted in higher levels of SFA and reduced ergosterol content after heat shock (35).

Variations in the number of genes encoding Δ9-FA desaturases have been observed across fungal organisms. However, the general trend shows that most fungal species possess a single or two copies of this gene (Table 1). Phylogenetic analysis of Δ9-FA desaturase homologs in major fungal pathogens reveals a distinct evolutionary separation between filamentous fungi SdeA and the Ole1 enzymes of yeasts. SdeA orthologs from filamentous fungi (e.g., *A. fumigatus*, *A. nidulans*, and *A. niger*) form a monophyletic clade with high bootstrap support (≥96), indicating a conserved function. Yeast Ole1 enzymes (e.g., *S. cerevisiae*, *C. albicans*, *C. auris*, and *C. parapsilosis*) cluster independently, while human SCD1 and SCD5 desaturases are grouped at the base of the tree as a phylogenetically distant outgroup (Fig. 1A). Biochemically, both SdeA and Ole1 are characterized by the presence of an integrated CytB5 domain, a structural feature that enables direct electron transfer within a single polypeptide chain.

As shown in Table 1 and Fig. 1A, some pathogenic fungi returned more than two predicted orthologs. However, in some cases, these candidates either lack a CytB5 domain or have not yet been investigated in detail, preventing us from confidently classifying them as Δ9-fatty acid desaturases. Within the genus *Aspergillus*, *A. flavus* appears to be an exception, as four orthologs were identified, each containing a predicted CytB5 domain (Table 1). Functional characterization is required to determine whether all these genes are involved in the biosynthesis of oleic and palmitoleic acids. This situation mirrors the complexity observed in other fungi, such as the nonpathogenic and oleaginous species *Mortierella alpina*, where gene copy number does not directly predict functionality. This fungus belongs to the subphylum of Mucoromycotina and has great importance as an industrial producer of PUFA, such as arachidonic acid (89). *M. alpina* encodes two Δ9-desaturase genes, *OLE1* and *OLE2,* that can complement the *S. cerevisiae OLE1* mutation, suggesting that both are functional in this filamentous fungus (90). Curiously, in *M. alpina*, a third gene encoding a fatty acid Δ9-desaturase, named Δ9-3, was identified, sharing the typical features of fungal membrane-bound desaturases, including transmembrane domains, conserved histidine boxes, and a C-terminal CytB5 domain. However, unlike *OLE1* and *OLE2*, Δ9-3 failed to complement the *OLE1* mutation in *S. cerevisiae*, suggesting a distinct substrate specificity (91).

*A. nidulans* is also an exception since it has two identified Δ9-FA desaturases, *sdeA* and *sdeB* (56), and both contain the CytB5 domain (Table 1). Deletion of both genes results in decreased UFA production and increased SFA accumulation. Interestingly, *A. nidulans* can tolerate the loss of *sdeA* or *sdeB* without UFA supplementation. *sdeA* is essential for fungal viability at both low and high temperatures, and *sdeB* is primarily required for optimal growth at lower temperatures. However, double *sdeA* and *sdeB* deletion is lethal (56). Functionally, the deletion of *A. nidulans sdeA* leads to upregulation of the FA synthase gene, *fasA*, resulting in an approximately 3.5-fold increase in SFA production compared to wild-type levels (56). Notably, treatment of *A. fumigatus* with transchalcone, an FasA inhibitor, specifically disrupts the ER localization of the SdeA protein. This finding suggests that *de novo* SFA synthesis perturbation affects SdeA function and accumulation, likely due to the reduction in the availability of its substrates (35). Importantly, this mislocalization effect of SdeA in the presence of transchalcone is not observed in other ER-resident proteins. These findings indicate a regulatory interplay between SFA synthesis and FA desaturation and provide a foundation for further exploration of its role in cellular adaptation to stress, hypoxia, and antifungal treatments.

In *A. fumigatus*, two studies identified the locus AFUB_091500 in the A1163 strain as the *sdeA^OLE1^* ortholog (35, 36). Interestingly, a search in FungiDB (https://fungidb.org/fungidb/app) of the *A. fumigatus* A1163 to look for the presence of an additional putative Δ9-FA desaturase (*sdeB*) returned the sequence encoded by the gene AFUB_090930 (Table 1). However, a close analysis of AFUB_090930 indicated that it is annotated as a pseudogene in the A1163 strain. Although this gene is transcribed, there is no evidence that this transcript is translated into an active Δ9-FA desaturase. In one of the studies, the authors were unable to generate translational epitope fusions of AFUB_090930, and targeted deletion of the retrieved AFUB_090930 sequence did not reveal any phenotypes related to vegetative growth or in the presence of all cell membrane-perturbing agents (35). However, in the *A. fumigatus* Af293 strain, the putative *sdeB* ortholog (Afu7g05350) is annotated as a functional but uncharacterized gene (Table 1), which, upon sequence analysis, retains the desaturase domain but lacks the CytB5 domain. Unlike mice, which possess four SCD isoforms, humans have only two loci encoding SCD1 and SCD5 and an additional locus encoding a processed, but inactive, pseudogene (92). A similar scenario may explain the presence of the *sdeB* pseudogene in *A. fumigatus* A1163 strain and possibly in other fungal organisms. Further analyses are required to elucidate whether *A. fumigatus* expresses one or two Δ9-FA desaturases and to assess the impact of a second gene copy on *A. fumigatus* biology. Systematic genomic analyses of multiple clinical isolates could provide insights into the capacity of fungal pathogens to generate palmitoleic and oleic acids via *sdeA* (and possibly *sdeB*), especially given that antifungal tolerance is altered when exogenous oleic acid is supplemented in the culture medium (36).

### The unique organization of fungal Δ9-fatty acid desaturases: new insights into cytochrome b5 domain

Although structural studies of mammalian SCD1 have provided valuable information into substrate recognition, enzyme activity, and kinetic properties (44–46), our understanding of the comprehensive mechanisms involved in the sequence of reactions that lead to the desaturation of C9 remains incomplete. This is particularly true in pathogenic fungi, where the biochemistry of these enzymes is poorly understood. Fungal ∆9-FA desaturases are notably different in sequence composition and structural organization from their mammalian counterparts. Unlike mammalian SCDs, which function as part of a ternary complex with CytB5 and cytochrome b5 reductase (CytB5R) (45, 46), fungal desaturases often exist as fusion proteins that incorporate a CytB5 domain within the same polypeptide chain (93).

Crystallographic and computational modeling studies have shown that mammalian SCD1 possesses four transmembrane helices and features a hydrophobic tunnel that facilitates substrate binding and desaturation. The active site includes three conserved histidine-rich motifs (HXXXXH, HXXHH, and QXXHH), comprising nine histidines that coordinate a nonheme di-iron center vital for catalysis (45, 46). These histidine-rich motifs are conserved in fungal homologs, including *A. fumigatus* (35).

Studies with rat and human SCD1 have revealed that this catalytic di-iron (Fe-Fe) center creates a unique environment featuring mixed coordination states, including a pentahistidine-coordinated FeA and a tetrahistidine-coordinated FeB (46). The complexity of the reaction mechanism has been addressed to emphasize the importance of the CytB5 domain (94). SCD1 employs an electron transfer system mediated by CytB5, wherein electrons from NAD(P)H are first transferred to CytB5R, a flavoprotein involved in multiple redox reactions. This process reduces the heme-iron domain of CytB5 protein. The reduced CytB5 then donates electrons sequentially to the di-iron center within the catalytic site of SCD1, allowing iron atoms to cycle between Fe(II) and Fe(III) states—a prerequisite for controlled O_2_ activation (Fig. 1B). The authors proposed that SCD1 follows a novel mechanism among di-iron enzymes, involving O_2_ binding at the active Fe(II)-Fe(II) center, forming a FeA(II)-•OH + FeB(II)-•OOH intermediate. Subsequently, proton and electron addition (facilitated by CytB5-derived electrons) yields FeA(II)-H_2_O, followed by oxygen bond dissociation, which releases the first water molecule and produces a unique triple-hydroxyl species (FeA(II)-•OH +FeB(II)-(•OH)_2_) (95). Internal hydrogen rearrangement within FeB(II)-(•OH)_2_ then leads to the creation of a high-valent FeB(IV)═O species, which abstracts hydrogen from C9 of the specific substrate—either palmitoyl-CoA or stearoyl-CoA—while FeA(II)-•OH abstracts hydrogen from C10, resulting in the final products: palmitoleic or oleic acids. Finally, during the restoration of the di-iron center, FeB(II)-•OH is converted into FeB(II)-(OH_2_)_2_ through an additional proton/electron input, releasing the second water molecule and thus completing the catalytic cycle. Thus, the CytB5R-CytB5 electron transfer system ensures a controlled and efficient desaturation process, highlighting the functional specialization of this pathway.

As aforementioned, fungal SCD1 homologs SdeA^Ole1^ are fusion proteins with the CytB5 domain positioned at the C-terminal region within a single polypeptide chain. The presence of a CytB5 domain found in fungi ∆9-FA desaturases was first identified during the early 90s for *S. cerevisiae* Ole1 (93); however, this feature has been overlooked in the biology of pathogenic fungi. The authors demonstrated that complementing an *OLE1* mutant with a rat SCD1 required the co-expression of a functional rat CytB5 gene. Consistently, targeted disruption of the CytB5 domain located at the C-terminal region of *OLE1* generated an auxotrophic strain for UFAs. This finding is remarkable because it indicates that other endogenous CytB5 proteins outside Ole1 cannot compensate for the specific function of the CytB5 naturally fused to Ole1 (93). Similarly, data from our laboratory indicate that deletion of the CytB5 domain in *A. fumigatus sdeA* is essential for viability (J. E. M. Campanella et al., unpublished).

Comparing these divergent modes of catalysis between mammals and fungi, it is tempting to speculate that the need for external CytB5 may add complexity to the mammalian systems as it requires precise spatial organization of SCD1, CytB5, and CytB5R in the membrane to ensure efficient electron transfer and catalysis (Fig. 1A). Additionally, mammalian SCD1 is more reliant on proper expression and interaction of multiple proteins, which can be affected by alterations in membrane composition or changes in protein-protein interactions, factors that are perhaps less of an issue for fungal desaturases with integrated CytB5, thus streamlining the catalytic process. As a result, the fungal enzyme is self-sufficient as it operates as a single multifunctional unit (Fig. 1B).

The divergence in the structural organization of fungal and mammalian Δ9-FA desaturases reflects their adaptation to different physiological needs. A past recombination event may explain the presence of a CytB5 domain in fungal Δ9 desaturases. It is plausible that this event occurred after the divergence of fungal and animal ancestors but before the diversification of present-day fungi, leading to the integration of an electron donor domain within the desaturase itself (96).

**Fig 2:**
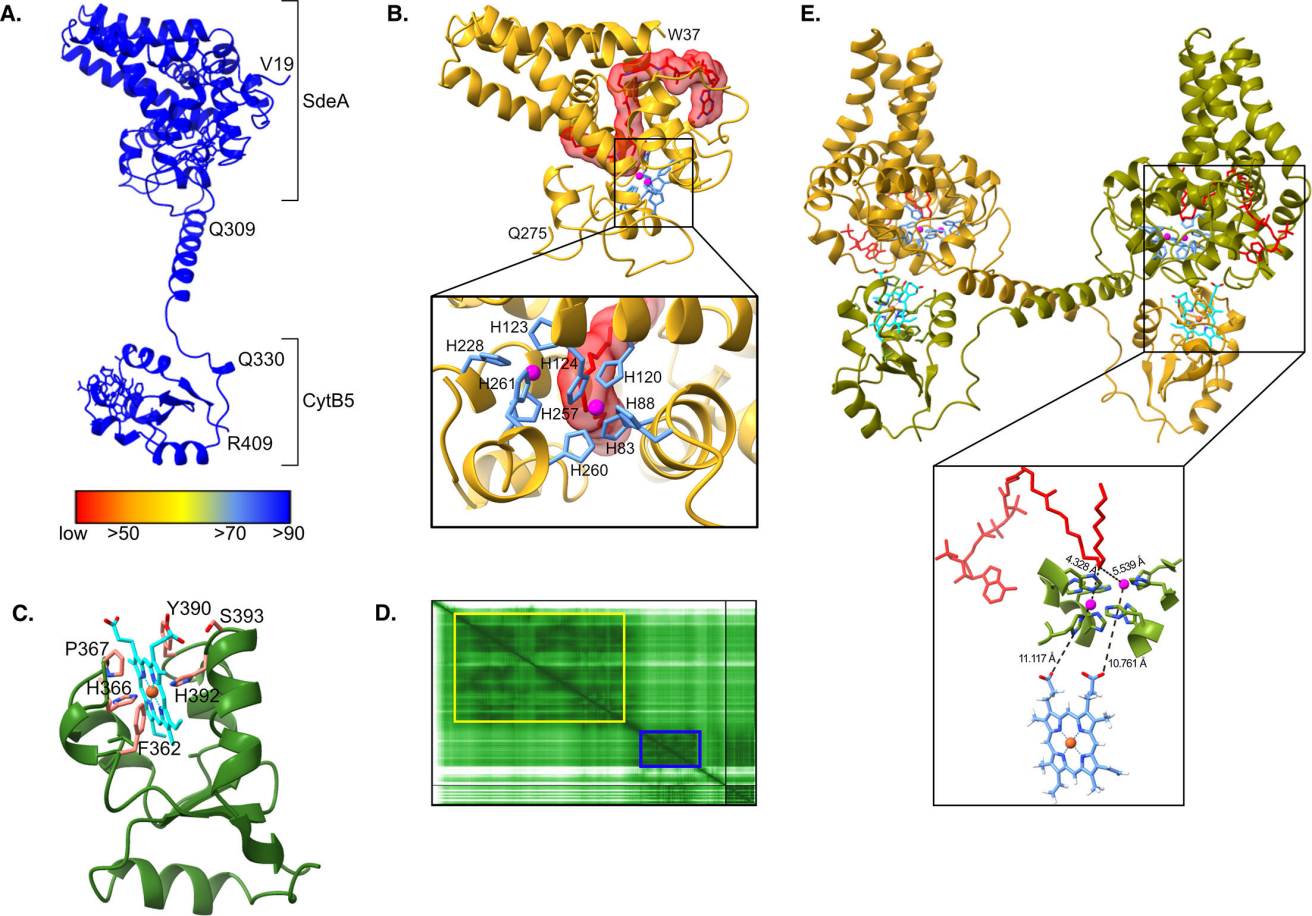
SdeA monomer from *A. fumigatus* predicted by Alphafold3. (**A**) SdeA structure (residues 19–409) colored by pLDDT confidence. The structure is composed of two domains: a desaturase domain (SdeA), ranging from residues 19 to 309, and a cytochrome b5 domain, ranging from residues 330 to 409. These domains are separated by a 21-residue-long linker. (**B**) Desaturase domain of SdeA (residues 37–275), colored in golden. Stearoyl-CoA molecule was docked in the structure by aligning SdeA against the human SDC1 crystal structure (PDB 4zyo). Stearoyl-CoA is depicted in red, with a 3 Å density around it. Iron ions are depicted in magenta. On the lower panel, the nine histidines belonging to three conserved histidine-rich motifs (HXXXXH, HXXHH, and QXXHH) in the catalytic site are depicted in blue, close to the aligned iron ions. (**C**) Cytochrome b5 domain of SdeA (residues 330–409), depicted in green. The heme prosthetic group is colored in cyan with oxygen atoms in red, nitrogen in blue, and iron in orange. (**D**) Predicted aligned error plot for SdeA monomer. Two clear domains are depicted, whereas the desaturase domain is highlighted in yellow, and the CytB5 domain is highlighted in blue. (**E**) SdeA dimer (residues 19–409), with protomer 1 depicted in gold and protomer 2 in green arranged in the stable dimeric structure. Stearoyl-CoA, depicted in red, was docked into the protomers by aligning SdeA against the human SDC1 crystal structure (PDB 4zyo). The heme prosthetic groups are depicted in cyan, while iron ions are depicted in magenta (SdeA domain) and red (heme). The lower panel shows the arrangement of the molecules involved in SdeA catalysis. Upon dimerization, the heme group of protomer 2 will be positioned in proximity to the desaturase catalytic site of protomer 1 and vice versa. Distances between the heme, the iron ions, and the docked stearoyl-CoA are depicted as black dotted lines. Heme group is colored in blue with oxygen atoms in red and iron in orange, stearoyl-CoA depicted in red, iron ions in SdeA are colored magenta, and SdeA residues in green and blue, where blue color indicates the nitrogen atoms of the coordinating histidines.

Using *A. fumigatus* SdeA as a model, we preliminarily investigate the structural and functional relationships of this fungal fusion enzyme. We performed *in silico* modeling using the AlphaFold3 server (94) with default parameters, including one heme group as ligand and two Fe^3+^ ions, and visualized the structures and error plots using ChimeraX (97). The full-length SdeA protein consists of two distinct domains connected by a long helical hinge region (Fig. 2A). The N-terminal domain (residues 19–309) showed a high predicted local distance difference test (pLDDT) score and significant structural similarity with the crystallized human SCD1 (PDB: 4ZYO), with a root mean square deviation (RMSD) of 0.8 Å over 204 pruned Cα atom pairs. The superposition of these structures revealed that the substrate stearoyl-CoA molecule, co-crystallized with human SCD1, aligned well within the predicted substrate-binding pocket of SdeA. Additionally, the two Fe^3+^ ions were predicted in the equivalent position of the two Zn^2+^ ions present in the PDB structure, coordinated by nine conserved histidine residues, supporting the hypothesis that the catalytic mechanism of human SCD1 could be extrapolated to its fungal homolog (Fig. 2B). It is important to note that the presence of zinc atoms in the di-iron pocket of the model may be an artifact from the deposited PDB structure, with iron likely being the native metal *in vivo*, as suggested by the authors of the human SCD1 crystallography (45, 98). Indeed, it has been demonstrated that in the presence of zinc, SCD1 is inactive, and the authors propose a method to replace zinc with iron in recombinant SCD1 (99).

The C-terminal domain (residues 330–409) showed structural similarity to CytB5, while residues 410–456 were predicted to have low pLDDT scores. Superposing the nuclear magnetic resonance (NMR) structure of rat CytB5 (100) (PDB: 1BFX) retrieved an RMSD of 1.0 Å over 59 pruned Cα atom pairs. Heme is coordinated in an equivalent position to CytB5, where histidines 366 and 392 serve as axial ligands for the heme iron center (Fig. 2C). These findings reinforce the notion that the SdeA C-terminal domain retains the fully functional electron transfer capability characteristic of CytB5.

Despite the fusion of the desaturase and the CytB5 domains shown by the primary sequence, the initial SdeA monomer model we generated did not suggest a direct interaction between the N-terminal desaturase and CytB5 domain, raising questions about the efficiency of intramolecular electron transfer in this putative fusion protein. In fact, the predicted aligned error plot of SdeA indicated low confidence in the spatial orientation between the N-terminal and C-terminal domains (Fig. 2D). At the same time, the helical hinge that apparently separates the two domains in the model did not favor interdomain interaction. Given that previous studies have suggested that the Ole1 activity may require dimerization for full functionality (99), we hypothesized that the observed domain separation might facilitate an intermolecular electron transfer mechanism upon dimerization in the fusion protein.

To further explore this possibility, we employed AlphaFold3 to generate a dimeric SdeA model. The resulting structure revealed a striking arrangement in which the C-terminal CytB5 domain of one protomer interacts with the N-terminal desaturase domain of the adjacent protomer, positioning the heme group in close proximity to the putative iron coordination site (Fig. 2E—upper panel). This topology mirrors the transconfiguration proposed for other desaturase systems (46) and provides a plausible structural basis for efficient electron transfer upon dimerization (Fig. 2E—lower panel).

These structural insights suggest that SdeA may have evolved a dimerization-dependent mechanism to optimize electron transfer and catalytic efficiency. This finding aligns with a recent biochemical study, demonstrating that mammalian SCD1 forms a stable ternary complex with CytB5 and CytB5R, which influences enzyme activity. Notably, when these mammalian proteins were artificially fused into a single polypeptide—mimicking the fungal enzyme architecture—electron transfer from CytB5 to the desaturase domain occurred more rapidly and efficiently than in a separate protein complex (44). This supports the idea that the unique fusion of a CytB5 domain to the C-terminus of the desaturase in fungi may have conferred an evolutionary advantage, enhancing their ability to adapt to environmental stresses rapidly. However, experimental validation is required to confirm this hypothesis and the structural model we propose here, which, to our understanding, unprecedentedly leverages the structure of all fungal Δ-FA desaturase enzymes.

### Δ9-Fatty acid desaturases inhibition and perspectives for fungal pathogens

Given the essential role of SdeA^Ole1^ in fungal lipid metabolism, the potential of Δ9-fatty acid desaturase inhibitors as antifungal agents deserves further investigation, especially since inhibitors of SCD1 have been extensively studied in human metabolic and oncologic disorders (reviewed in references 101–103). Several SCD1 inhibitors have already been tested in preclinical and clinical studies, demonstrating promising pharmacological profiles. Examples of mammalian inhibitors include MK-8245 (type 2 diabetes) (104, 105) and aramchol (nonalcoholic steatohepatitis) (106). Additionally, a tumor-specific SCD1 prodrug was shown to suppress tumor growth in cancer cell lines (107). These findings highlight the diverse therapeutic potential of SCD1 inhibitors and suggest that they could be repurposed for antifungal applications in the future. Since SCD1 knockout mice exhibit relatively mild phenotypes (108), these inhibitors could provide selective antifungal effects with minimal host toxicity. Furthermore, combining these inhibitors with conventional antifungals might enhance their efficacy, potentially overcoming resistance mechanisms and expanding current treatment options or preserving the clinical utility of existing drugs.

Recent studies highlight the potential of targeting Δ9-FA desaturase in clinically relevant fungal pathogens. DeJarnette et al. (34) performed *C. albicans* whole-cell screening of compounds affecting FA synthase or desaturase activity. Their study identified four acyl hydrazides as promising candidates for Ole1 inhibition, showing broad-spectrum antifungal activity against *C. albicans*, *C. auris*, Mucorales, and *A. fumigatus*. In *C. albicans*, their efficacy was reduced upon supplementation with UFAs, suggesting an action on Ole1. However, their direct inhibition of Ole1 was not confirmed (34). In addition, a recent discovery identified aryl-carbohydrazide analogs as potent inhibitors of Ole1 in *C. auris* and *C. albicans* (109). Through chemical library screening, SPB00525 [N′-(2,6-dichlorophenyl)-5-nitro-furan-2-carbohydrazide] compound was identified as capable of reducing UFA and increasing SFA content in *C. albicans*. It exhibits strong antifungal activity against multiple *C. albicans* strains resistant to standard antifungals, inhibiting invasive hyphal growth, biofilm formation, and attenuating virulence in *Galleria mellonella* infection model (109).

Additionally, MBX-7591, a nonspirocyclic piperidine molecule, has been recently identified as a potentiator of contemporary triazole antifungals (71). MBX-7591 enhances the efficacy of triazoles against drug-resistant strains, such as *A. fumigatus*, Mucorales, and *C. neoformans*. The mode of action of MBX-7591 involves the inhibition of the conversion of SFA to UFA, thereby affecting fungal membrane integrity (71). However, how this molecule structurally interacts with SdeA to lower the levels of oleic acid remains to be defined. Interestingly, MBX-7591 exhibits inhibitory activity against human cytochrome P450 enzyme CYP3A4 in the clinical tests. It will be interesting in future studies to interrogate the potential activity of this molecule on SdeA or other cytochrome-containing enzymes.

Natural products have also been explored as SCD inhibitors, showing potential for antifungal applications. Cyclopropenoid fatty acids, for example, have long been known to inhibit desaturases both *in vitro* and *in vivo* in mammalian models (110). Sterculic acid, a cyclopropane FA originally found in the seeds of *Sterculia foetida*, inhibits mammalian SCD due to its highly strained and reactive propene ring and a double bond located exactly between C9 and C10 in its structure (111). This compound has been investigated as an adjuvant therapy for diseases, such as nonalcoholic steatohepatitis, Alzheimer’s disease, cancer, and retinal disorders. Sterculic acid derivatives have been tested against some human pathogens, such as *Toxoplasma gondii* (112) and *Plasmodium falciparum* (113), suggesting that these compounds could also inhibit fungal Δ9-FA desaturases. Another promising natural product tested for antifungal activity through Δ9-desaturase inhibition is (Z)-14-(furan-2-yl)-tetradeca-9-en-11,13-diynoic acid (EV-086), obtained from plant cell extracts of *Anarrhinum bellidifolium* (33). This small molecule acts on Δ9-FA desaturation by targeting membrane phospholipids and influencing *OLE1* transcription, which is strongly upregulated in the presence of EV-086. The compound increased the SFA/UFA ratio, suggesting effective action on Ole1. However, EV-086 was ineffective in rat and mouse models of systemic candidiasis (33) but impaired the growth of the dermatophyte *Trichophyton mentagrophytes*, indicating that its mechanism of action in other fungi requires further investigation. Additionally, computational approaches have been used to identify natural compounds with potential inhibitory activity against SCD1; however, these candidates have not yet been validated *in vivo* (114).

### Future perspectives for targeting fungal desaturases

As aforementioned, the lack of a defined structure for SdeA/Ole1 compromises the discovery of specific inhibitors. Unfortunately, direct binding of molecules that have been identified as inhibitors of human or fungal Δ9-FA desaturases has not been demonstrated so far. This reinforces the importance of studying the structural and biochemical aspects of this desaturase. The unique organization of fungi into a self-sufficient fusion protein comprising the desaturase and CytB5 domain protein raises the intriguing possibility of directly inhibiting the CytB5 domain itself, thereby disrupting membrane fluidity through alterations in the SFA/UFA ratio. Targeting CytB5 proteins in fungi could offer a novel strategy for inhibiting desaturase activity, potentially impairing fungal growth without affecting the host. To assess potential off-target effects of compounds targeting the CytB5 domain of SdeA, we performed a BLASTp analysis using the C-terminal CytB5 domain (Leu331-Arg409) as a query against the *A. fumigatus* genome, which identified 15 proteins with moderate sequence identity (36%–43%) and *e*-values ranging from 7e−08 to 6e−04. Most of these proteins are annotated as having predicted heme-binding properties, oxidoreductase activity, and roles in redox-related metabolic processes. Not all of them are directly involved in lipid metabolism but rather in different metabolic reactions, suggesting functional diversity among CytB5-containing proteins. In addition, several of these proteins remain uncharacterized. A similar search using the rat CytB5 (employed in the structural prediction shown in Fig. 2) domain retrieved 17 proteins with 28%–50% identity, again suggesting low conservation and the presence of diverse cytochrome subtypes. These suggest that selective inhibition of SdeA may be achievable, provided sufficient compound specificity that would minimize off-target effects. Developing new drugs or repurposing existing ones to target the CytB5 domain could be evaluated in combination with existing antifungal therapies to enhance their effectiveness. This opens new avenues for antifungal chemotherapy targeting the fungal cell membrane.

Furthermore, our structural modeling suggests that SdeA functions as a dimer, which requires further *in vivo* evaluation in *A. fumigatus*. To test this hypothesis, site-directed mutagenesis could be used to identify residues essential for dimerization, while structural approaches such as small angle X-ray scattering (SAXS), crystallography, or cryogenic electron microscopy (cryo-EM) could elucidate the conformation and interaction dynamics, providing insights into rational drug design targeting the catalytic site of the desaturase or the CytB5 electron transfer activity.
